# Impact on the Utilization of Reproductive, Maternal, Newborn and Child Health Care Services at Primary Health Care Level During First Wave of COVID-19 Outbreak in Pakistan

**DOI:** 10.7759/cureus.17430

**Published:** 2021-08-25

**Authors:** Ayaz A Baloch, Nelofer Baig, Fozia Baloch, Zamir Suhag

**Affiliations:** 1 Research, People's Primary Healthcare Initiative (PPHI) Sindh, Karachi, PAK; 2 Pediatrics and Child Health, Liaquat University of Medical and Health Sciences, Jamshoro, PAK

**Keywords:** covid-19, primary health care, pandemic, rmnch, mnch

## Abstract

Introduction: Very little is known and predictable on how long the COVID-19 pandemic will last, even though dramatic preventive measures to flatten the curve and stop its transmission have been taken worldwide periodically since its start. These preventive measures coupled with people’s fear of COVID-19, significantly altered people’s health-seeking behavior and healthcare accessibility. This study aims to measure the impact of the COVID-19 pandemic on the utilization of reproductive, maternal, newborn, and child health (RMNCH) care services at primary health care (PHC) facilities in Pakistan.

Methods: A retrospective study was conducted in 22 districts using data from 1169 primary health care facilities. The District Health Information System (DHIS) was used to retrieve district-wise services utilization data from January to April 2020 i.e. January to February 2020 (pre-COVID-19) and March to April 2020 (during COVID-19). The monthly difference (in percentage change) for selected RMNCH services indicators was calculated using the expected number of clients (January to February average) versus the actual number of clients for March and April 2020.

Results: We observed a decrease of 12.5% for March and 33% for April 2020 in the total number of clients who availed of primary health care services in Sindh as compared to the pre-COVID-19 monthly average. A decrease in first antenatal care visits (15.6% and 16.3%), delivery coverage (12.8% and 17.9%) and family planning client visits (31.6% and 36.3%) were observed for March and April 2020 respectively. The pentavalent vaccine results revealed a decrease of 19.3% and 63.1%, while a decrease of 17.3% and 54.3% were observed for children receiving the first dose of measles vaccine in March and April, respectively.

Conclusions: Our findings show that the number of clients who avail of routine care or RMNCH services at the primary health care level considerably declined during the initial phase of the first wave of the COVID-19 outbreak in Sindh, Pakistan. These results highlight a rising threat to poor RMNCH and outcomes.

## Introduction

In December 2019, the first outbreak of coronavirus diseases (COVID-19) originated in the city of Wuhan, Hubei province of China [1]. It quickly spread worldwide, becoming a pandemic in no time, resulting in nearly 200 million confirmed cases and 4.2 million deaths by the end of July 2021 [2].

COVID-19 is a highly infectious disease but has low mortality compared to other coronaviruses [3]. It is generally believed to spread from person to person and has a high infectivity rate [4]. In the wake of this growing outbreak, COVID-19 has received vast global scientific attention to investigate its aetiology, modes of transmission, potential treatment protocols, prognosis, effective prevention measures, and vaccine development since its first outbreak in Wuhan, China. The international and national public health agencies worldwide advised people to adopt preventive measures in the wake of this growing pandemic, such as wearing masks in public, maintaining social distancing, and performing frequent hand hygiene [4]. 

In Pakistan, the first laboratory-confirmed COVID-19 case was notified from Karachi on February 26, 2020 [5]. As of August 12, 2021, there were 1,085,294 laboratory-confirmed COVID-19 cases and 24,187 deaths reported in Pakistan [5]. Karachi, a densely populated city of Pakistan, contributed to nearly 30% of the total COVID-19 confirmed cases in the country [5]. Given the rising number of cases of COVID-19 among travellers returning from hard-hit countries, the provincial government of Sindh announced a lockdown across the province and the closure of outpatient departments (OPDs) in major public hospitals across the province from March 24 till May 22, 2020 [6]. Consequently, most private-sector healthcare providers (HCP) in the province closed their non-emergency and routine outpatient care services [7]. 

Pakistan falls into low and middle-income countries (LMIC), and its maternal and child health (MNCH) indicators are not very encouraging. The most recent Pakistan Demographic and Health Survey (PDHS) conducted between 2017 to 2018 reported reproductive, maternal, neonatal, and child health (RMNCH) indicators below acceptable levels [8]. In rural Sindh specifically, the contraceptive prevalence rate (CPR) was estimated as low as 21.4%, only 41% of pregnant women received four or more antenatal care (ANC) visits, and only 58.2% of deliveries were conducted in a health facility [8].

People's Primary Health Care Initiative (PPHI) Sindh, a non-governmental not-for-profit organization, currently holds a significant share in public primary health care (PHC) facilities in Sindh under public-private partnership (PPP) with the provincial government since 2007. It currently manages more than 60%, i.e., 1232 state-owned PHC facilities in Sindh. Most of the PPHI Sindh managed health facilities (HF) are located in rural areas of Sindh. Since its inception and gradual expansion in its share of PHC facilities, RMNCH services coverage indicators have improved significantly in the province's rural areas. In a community-based survey conducted by PPHI Sindh in 2019, RMNCH indicators such as ANC, CPR, institutional delivery, and postnatal care (PNC) in PPHI Sindh health facility catchment areas were found to be noticeably better than catchment areas of the Department of Health (DoH) managed primary health care facilities [9]. However, further improvement is required to provide quality and accessible PHC services in the province.

Furthermore, with the ongoing COVID-19 crisis, the rural areas of Sindh are at high risk as PHCs have reported a notable reduction in the number of patients accessing HFs. In Italy, a study reported delays in service provision and access to health care hampered due to mobility restrictions and fear of contagion [10]. People's extreme fear of becoming infected with COVID-19 is associated with its novelty, high community and nosocomial transmission [11]. Very little is known and predictable to understand how long the outbreak will last even though dramatic preventive measures to flatten the curve and stop its transmission have been taken worldwide. Unfortunately, the consequences of the COVID-19 pandemic are not limited to only those who are infected. Its impacts on the community's healthcare-seeking behaviour and accessibility to medical services are enormous and must not be overlooked.

Therefore, it is critical to determine the extent to which the COVID-19 pandemic and subsequent preventive measures have altered healthcare-seeking behaviours and limited the accessibility to PHC services. This paper has assessed the utilization of RMNCH services at the PHC level during the first wave of the COVID-19 outbreak in Sindh, Pakistan.

## Materials and methods

The study used a retrospective assessment of RMNCH services indicators to assess how the COVID-19 outbreak impacted public health-seeking behavior and accessibility to PHC services. RMNCH coverage indicators from the list of 100 core global services coverage indicators of the World Health Organization (WHO) were selected in this study. We retrieved the data on RMNCH service indicators from 1238 health facilities in 22 PPHI Sindh working districts for the first four months of the year 2020, i.e., January and February (pre-COVID-19 period), March and April (during the COVID-19 outbreak period). PPHI Sindh does not operate any HFs in six districts of the Karachi region. Furthermore, data from 55 HFs of the district of Shaheed Benazir Abad was not available as PPHI Sindh took over the management of these facilities only in March 2020. Moreover, data from 14 HFs were excluded due to missing information for any study indicator in any month of the study period. Finally, the data from 1169 health facilities from 22 districts were used in the analysis.

Data for selected RMNCH indicators, both before the pre and ongoing COVID-19 outbreak period, was retrieved from the District Health Information System (DHIS) dashboard. We collected monthly data on the total number of clients visiting PPHI Sindh HFs during the study period for any purpose (OPD visits), the number of pregnant women receiving their first antenatal care visit (ANC-1), the total number of pregnant women receiving their first or follow up antenatal care visit (ANC all visits), number of pregnant women receiving the second dose of tetanus toxoid (TT) vaccine during their ANC visits, number of normal vaginal deliveries (NVDs) conducted at PPHI Sindh HF in a month, number of under-one-year-old children receiving their scheduled third dose of pentavalent vaccine (Penta III), number of under-one-year-old children receiving their scheduled first dose of measles vaccine (Measles-1), number of clients visiting family planning services (FP clients) and number of clients using laboratory services (lab investigation). Furthermore, we also analyzed administrative data according to disease incidence reports for common acute and chronic illnesses for January, February, March, and April 2020.

We calculated the district-wise average from January to February 2020 (pre-COVID-19) against each indicator and used it as the expected value for comparison with observed values. Differences (in percentage change) between observed and expected values for March and April 2020 (during COVID-19) were calculated to determine whether and to what extent changes in services utilization were observed during the COVID-19 outbreak. We used thematic maps constructed with the help of ArcGIS Desktop version 10.6 (Environmental Systems Research Institute; Redlands, CA ) to show the district-wise percentage change in OPD visits during March and April 2020.

## Results

A total of 3,510,412 beneficiaries visited PPHI Sindh HFs for the first two months of the year 2020, i.e., before the start of the COVID-19 outbreak in Pakistan. During the initial COVID-19 outbreak (March to April 2020), a cumulative decrease of 11.8% and 32% in patients accessing PPHI Sindh HFs was observed, respectively. Among overall outpatient department (OPD) visits, on average 53.6 % were female clients with minimal variation (53% to 54%) throughout the first four months of 2020. An overall decrease in pregnant women seeking first-ever antenatal care visit after conception i.e., ANC-1 (15.6% and 16.3%), pregnant women receiving the second dose of TT vaccine during pregnancy i.e., TT-2 (6.5% and 28.3%), number of NVDs conducted at PPHI HFs (19.6% and 28.9%) and family planning (31.6% and 36.3%) services were observed for March and April respectively.

Moreover, a noticeable decrease in children receiving their scheduled vaccination was observed during the first two months of the COVID-19 outbreak in Sindh. The number of children receiving the Penta-III vaccine as per their expanded program on immunization (EPI) schedule decreased by 19.3% and 63.1% for March and April, respectively. Simultaneously, the trends showed a similar pattern for the Measles-1 vaccine, which observed a decrease of 17.3% and 54.3%. 

**Figure 1:**
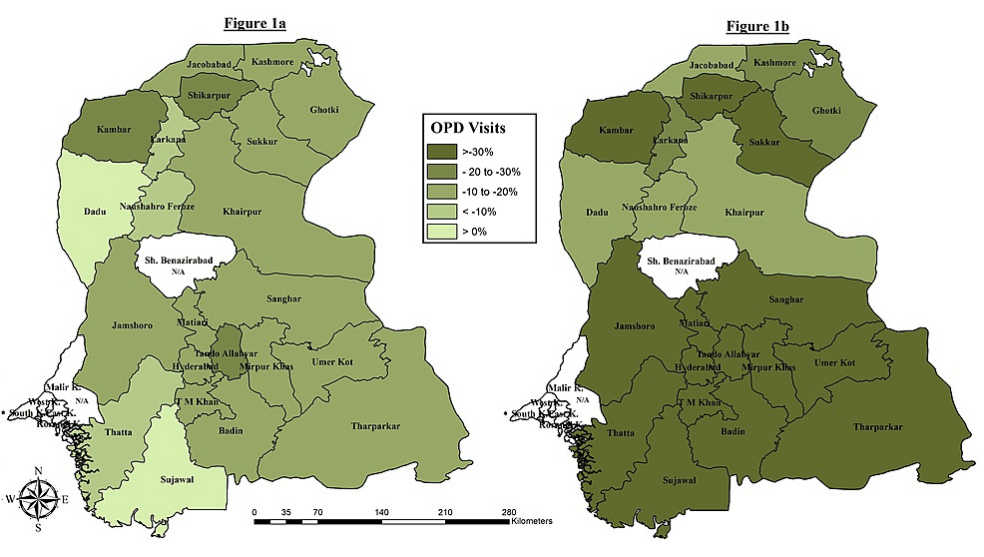
Thematic map illustrating district-wise percentage change in the number of clients availing primary health care services (OPD visits) for March 2020 (Figure 1a) and April 2020 (Figure 1b) with respect to the average of January and February 2020 (pre-COVID-19). OPD: Outpatient Department; N/A (Data): Not Available Source: Author's own work using open-source ArcGIS Desktop version 10.6

The results for March 2020 showed a negative impact in OPD turnover in almost all districts except for two districts, i.e., Dadu (0.1% increase) and Sujjawal (1.4% increase) as seen in Figure 1a. Whereas a significant decline in the number of OPD visits was observed at all PPHI Sindh working districts for April 2020, as depicted in Figure 1b. The highest decrease in the outpatient turnover was observed in districts of Tando Allah Yar (71%) and TM Khan (54.2%) (Figure 1b).

**Figure 2:**
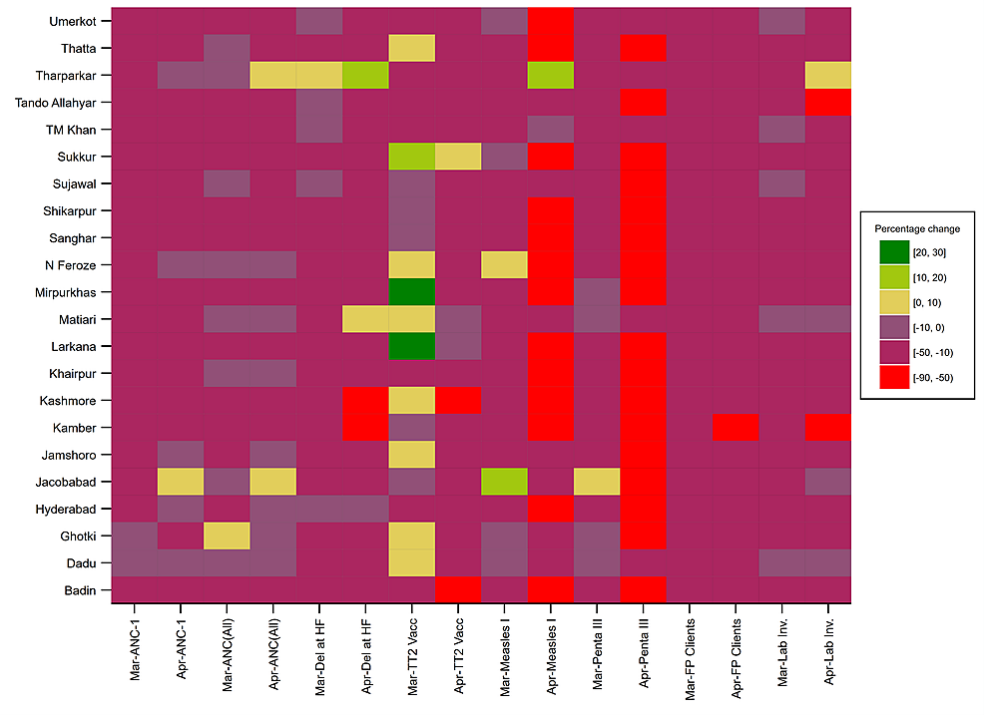
Heat map representing district-wise percentage change in utilization of RMNCH services during March and April 2020

We observed a declining trend in the number of clients accessing primary care facilities to avail of different services during the initial outbreak period for almost all indicators in all districts (Table 1) (Figure 2). The results reveal a higher decline in utilization of services for April than in March 2020. Furthermore, the highest decrease was observed for indicators related to childhood immunization.

**Table 1:** Indicator-wise difference in percentage change for March and April 2020 with respect to the average of January and February in 22 PPHI Sindh working districts of Sindh ∆: Percentage change; ANC:  Antenatal care; Vacc: Vaccine; TT2: Second dose of toxoid tetanus; Lab: Laboratory; FP: Family planning; TM Khan: Tando Muhammad Khan

District	ANC-1		ANC (All visits)		Delivery Coverage		TT2 Vacc		Measles I Vacc.		Pentavalent III Vacc.		FP Clients		Lab Investigations	
	∆ March	∆ April	∆ March	∆ April	∆ March	∆ April	∆ March	∆ April	∆ March	∆ April	∆ March	∆ April	∆ March	∆ April	∆ March	∆ April
Badin	-17.3%	-12.2%	-13.7%	-15.4%	-18.1%	-21.2%	-24.7%	-64.7%	-16.8%	-84.7%	-14.8%	-77.5%	-32.0%	-40.4%	-15.6%	-19.9%
Dadu	-6.7%	-6.5%	-2.2%	-6.2%	-33.0%	-45.6%	0.3%	-15.5%	-4.6%	-17.1%	-4.6%	-30.3%	-34.0%	-32.1%	-4.2%	-6.5%
Hyderabad	-19.8%	-5.5%	-13.5%	-5.6%	-3.0%	-2.2%	-26.3%	-46.1%	-14.7%	-60.4%	-28.4%	-67.5%	-33.7%	-38.7%	-19.5%	-16.0%
Sujawal	-14.0%	-16.8%	-3.4%	-10.5%	-0.2%	-22.3%	-0.1%	-42.2%	-27.2%	-49.3%	-18.3%	-51.1%	-31.1%	-39.5%	-4.4%	-20.6%
Jamshoro	-14.0%	-6.1%	-11.6%	-7.0%	-17.6%	-26.9%	9.0%	-20.7%	-17.1%	-47.4%	-20.9%	-60.8%	-33.5%	-35.8%	-15.0%	-16.1%
Tando Allahyar	-32.7%	-47.7%	-25.1%	-46.6%	-4.8%	-14.0%	-16.9%	-31.6%	-39.9%	-38.6%	-38.6%	-70.1%	-38.8%	-39.1%	-19.2%	-60.9%
Thatta	-10.1%	-11.5%	-9.2%	-11.8%	-14.8%	-27.4%	4.4%	-38.7%	-34.2%	-70.0%	-23.7%	-59.3%	-37.8%	-48.4%	-17.1%	-21.1%
Matiari	-14.4%	-11.2%	-5.8%	-9.8%	-18.4%	0.4%	3.9%	-1.7%	-17.5%	-49.0%	-3.5%	-46.3%	-23.8%	-31.8%	-0.6%	-9.6%
TM Khan	-15.6%	-22.0%	-14.7%	-22.0%	-9.0%	-13.7%	-19.2%	-17.4%	-27.1%	-7.8%	-34.2%	-33.5%	-37.8%	-40.4%	-8.3%	-33.7%
Jacobabad	-12.5%	0.8%	-7.9%	8.6%	-24.5%	-48.0%	-0.4%	-16.7%	15.1%	-46.7%	7.1%	-54.8%	-33.1%	-33.0%	-14.2%	-4.0%
Larkana	-10.2%	-14.4%	-13.5%	-22.8%	-28.6%	-45.2%	27.5%	-5.6%	-34.6%	-70.4%	-21.7%	-89.0%	-29.2%	-38.5%	-14.8%	-34.6%
Shikarpur	-16.7%	-24.5%	-19.6%	-20.8%	-28.5%	-42.4%	-7.5%	-18.6%	-39.9%	-70.4%	-25.9%	-57.9%	-39.5%	-35.9%	-18.7%	-29.3%
Kamber	-21.3%	-46.2%	-16.5%	-46.0%	-25.4%	-53.7%	-6.0%	-33.8%	-22.4%	-64.4%	-23.3%	-73.9%	-48.0%	-63.8%	-24.6%	-57.7%
Kashmore	-14.7%	-19.1%	-11.1%	-17.1%	-27.7%	-54.7%	1.4%	-51.8%	-18.7%	-73.3%	-10.7%	-73.2%	-42.7%	-50.0%	-23.7%	-27.7%
Khairpur	-14.3%	-15.7%	-5.1%	-4.6%	-22.3%	-45.6%	-21.8%	-41.4%	-28.2%	-66.0%	-27.6%	-75.0%	-29.0%	-25.5%	-18.2%	-35.1%
N Feroze	-12.4%	-9.7%	-6.7%	-7.8%	-20.7%	-31.7%	2.7%	-22.1%	0.9%	-66.3%	-24.7%	-75.3%	-33.6%	-26.8%	-12.4%	-13.7%
Sukkur	-21.9%	-17.5%	-18.2%	-19.3%	-28.0%	-32.2%	16.0%	3.8%	-8.8%	-60.1%	-12.0%	-57.9%	-34.9%	-41.6%	-11.2%	-26.6%
Ghotki	-5.6%	-10.7%	3.5%	-1.9%	-25.2%	-33.2%	5.4%	-13.1%	-2.7%	-49.8%	-4.7%	-69.3%	-30.9%	-35.4%	-10.7%	-22.5%
Mirpurkhas	-18.4%	-27.2%	-14.9%	-23.8%	-13.6%	-21.3%	20.8%	-21.3%	-11.3%	-66.0%	-7.8%	-59.1%	-24.9%	-30.4%	-20.4%	-34.7%
Sanghar	-21.0%	-28.3%	-13.6%	-26.6%	-14.5%	-13.0%	-8.2%	-34.4%	-18.5%	-80.0%	-27.8%	-75.9%	-29.5%	-33.0%	-18.8%	-23.7%
Tharparkar	-11.9%	-1.5%	-4.7%	0.0%	2.9%	10.3%	-13.9%	-16.1%	-20.2%	15.7%	-27.7%	-33.2%	-28.2%	-32.5%	-11.6%	3.5%
Umerkot	-17.3%	-18.4%	-19.6%	-25.3%	-3.9%	-14.9%	-11.1%	-39.3%	-9.7%	-54.9%	-23.0%	-40.3%	-15.8%	-33.8%	-8.1%	-26.6%

## Discussion

The COVID-19 outbreak started in Pakistan in March 2020, and it is still very unclear how long this high transmission will persist given factors such as community unawareness, poverty, ineffective lockdown, and health illiteracy. In the given circumstances and keeping in view the existing situation, it is difficult to estimate when the outbreak will decline. However, during the COVID-19 pandemic so far, declining healthcare-seeking trends have been observed across the globe. A study conducted in a psychiatry hospital in Germany measured a 26.6% decrease in patients accessing emergency mental health services [12]. Another study from Beijing, China, evaluated the dental emergency centre trends and observed a decline of 38% [13].

Similarly, our study has shown a severe impact on overall services utilization, mainly RMNCH services at the PHC level in rural areas. While a decrease in OPD turnover was expected due to factors such as fear and stigma of getting infected, mobility restrictions during the lockdown, and due to public health authorities' prevention campaigns to refrain from unnecessarily visiting hospitals and clinics during the initial outbreak period; a remarkable decrease in institutional deliveries and decrease in children receiving their scheduled immunization is worrisome and alarming for all stakeholders. It is unknown whether these missed deliveries took place at homes or some public or private HFs. It is essential to mention here that most of the primary health care facilities are located in hard-to-reach areas in remote settings, and the literacy rate in these catchment populations is only under 40% [9]. Keeping in view the general fear of the public for going out of homes during this COVID-19 pandemic, particularly to hospitals and clinics, which are categorized as high risk for transmission, we hypothesize that most of these deliveries that failed to access health facilities perhaps had been conducted at homes through untrained traditional midwives. This increases maternal and neonatal health risk as institutional delivery is an essential indicator for achieving Sustainable Development Goal (SDG) for health.

It is still unknown how far these RMNCH services utilization trends will decrease at this outbreak's peak. If the observed trends continue to decline as evidenced in this study and do not improve to pre COVID-19 levels shortly, the already marginalized and underprivileged population of Sindh might face catastrophic health outcomes as evidenced by significant decreases in PHC seeking patterns. Unfortunately, these trends will result in complicated pregnancies due to lack of proper antenatal care, an increase in the number of home deliveries, and a surge in vaccine-preventable disease outbreaks in children under the age of five, and overall damage to RMNCH in already underprivileged and economically deprived areas.

The results strongly suggest that the fears of the COVID-19 pandemic noticeably influenced people's care-seeking behavior and significantly compromised people's accessibility to necessary health care services. Although most of the global attention is focused on the direct impacts of COVID-19, this study provides a glimpse of the extent of damage the COVID-19 pandemic is indirectly causing on people's ability to access PHC in LMIC countries, especially in the area of RMNCH. The healthcare-seeking patterns presented here may provide concerned authorities with a more comprehensive picture of the overall health impacts of the COVID-19 pandemic to guide public health officials to prevent avoidable health consequences due to fears of COVID-19 among the general population.

## Conclusions

The study analyzed PHC seeking patterns amid the COVID-19 outbreak in Pakistan to provide a baseline for future studies concerning how and to what extent the health of the public is being affected by this pandemic. Our findings are noteworthy in that we propose that COVID-19 pandemic health impacts go beyond those who are being infected. The associated dismay, terror, consternation have significantly changed general populations' care-seeking behaviors and are jeopardizing the health status of rural communities and the utilization of essential health services as well as access to PHC services in many areas. It is possible to safeguard and strengthen community health resilience by fighting this pandemic, but knowing the risks and vulnerabilities in the general population is key. Scaling community awareness campaigns by focusing on necessary preventive techniques and addressing myths and unnecessary fears will play a key role in restoring normal demand for PHC services in rural communities. Furthermore, holistic, evidence-based decision-making is the most important factor in such a situation to prioritize the preparedness and response strategies at all levels and limit the damages caused by public health emergencies.
